# Bacteriophage therapy created the necessary conditions for successful antibiotic suppression in a periprosthetic hip joint infection: a Case Report

**DOI:** 10.3389/fmed.2025.1564369

**Published:** 2025-04-25

**Authors:** Peter Wahl, Michel Schläppi, Archana Loganathan, Ilker Uçkay, Sandro Hodel, Benjamin Fritz, Jens Scheidegger, Sarah Djebara, Lorenz Leitner, Shawna McCallin

**Affiliations:** ^1^Division of Orthopaedics and Traumatology, Cantonal Hospital Winterthur, Winterthur, Switzerland; ^2^Faculty of Medicine, University of Bern, Bern, Switzerland; ^3^Department of Neuro-Urology, Balgrist University Hospital, University of Zurich, Zürich, Switzerland; ^4^Unit of Clinical and Applied Research, Infectiology, Balgrist University Hospital, University of Zurich, Zürich, Switzerland; ^5^Department of Orthopedics, Balgrist University Hospital, University of Zurich, Zürich, Switzerland; ^6^Department of Radiology, Balgrist University Hospital, University of Zurich, Zürich, Switzerland; ^7^Center for Infectious Diseases, Queen Astrid Military Hospital, Brussels, Belgium

**Keywords:** periprosthetic joint infection, PJI, bacteriophages, antibiotic suppression, *Staphylococcus aureus*, MRSA

## Abstract

**Introduction:**

Treatment failure remains an issue in periprosthetic joint infection (PJI). Bacteriophages offer new treatment options. However, there is still a lack of evidence to better define their usefulness and administration. We report a case in which antibiotic suppression was successful only after administration of bacteriophages.

**Case description:**

Antibiotic suppression was the only option for a 94-year-old male with methicillin-resistant *Staphylococcus aureus* (MRSA) PJI of the hip and of the knee. As the hip PJI could not be suppressed adequately, bacteriophages were administered locally and systemically together with daptomycin. This combined approach led to sufficient clinical improvement for further oral antibiotic suppression, although without infection eradication.

**Conclusion:**

The administration of bacteriophages may be a valuable, less-invasive adjunct therapy to successfully suppress PJI. Bacteriophage selection, preparation and administration, however, remains associated with administrative obstacles, greatly limiting availability and practicability. Nevertheless, research and developments in this domain should be pursued, particularly considering issues with future antibiotic limitations and cost associated with treatment failure in PJI.

## Introduction

Periprosthetic joint infection (PJI) remains a severe complication of arthroplasty, associated with a relevant morbidity and mortality (1–6). Despite many advances and standardizations in treatment, therapy failures remain a frequent issue, particularly when treatment options are limited by surgical aspects or by comorbidities (5, 7–11). Long-term antibiotic suppression therapy remains an option in case of failure to eradicate PJI (9, 12, 13).

However, prolonged administration of antibiotics is associated with a relevant rate of adverse reactions, and suppression of the infection may not be successful (4, 12, 13). Bacteriophages offer new treatment options, particularly considering increasing evidence for a synergistic effect of bacteriophages and antibiotics (14–17). Though, there is still a lack of evidence to better define their appropriate use and therapeutic role of their administration as well as the posology in musculoskeletal infections (14, 15). In selected cases where curative surgery is not feasible, and infection persists despite standard treatment, bacteriophage therapy may offer a viable adjunctive option, particularly when microbiological data support its use (15, 17).

We report such a case in which the last option of suppressive antibiotic therapy was successful only after administration of bacteriophages.

## Case description

A 94-year-old male with a complex medical history was hospitalized due to sepsis and new onset right hip pain. A therapeutic oral anticoagulation with apixaban had been maintained since a pulmonary embolism one year prior, and the patient suffered from chronic renal impairment (estimated clearance between 30 and 35 mL/min/1.7 m^2^). Total hip arthroplasty (THA) had been performed 7 years and medial unicompartimental knee arthroplasty had been performed 14 years before, respectively, both on the right side. Since THA, the patient had been operated repeatedly on his lumbar spine. Because of the pain irradiating to the right hip, aspiration of the hip joint had been performed 1.5 years after THA, excluding PJI, based on a low cell count and sterile microbiologic workup.

The patient had undergone lumbopelvic stabilization surgery two years earlier. Since surgery, he had not regained the ability to walk, but he remained pain-free and was able to manage most aspects of personal hygiene independently. Separately, due to an oncologic subtotal penectomy performed 12 years earlier, permanent bladder drainage had been necessary. In recent years, he had received multiple courses of antibiotics due to recurrent urinary tract infections, a potential source for the selection of multidrug-resistant microorganisms.

Following the patient’s request for therapeutic support, hemodynamic stabilization was performed, and an empiric parenteral antibiotic therapy with cefepime was introduced. Blood cultures returned positive within hours for *Staphylococcus aureus*, which was identified the next day as being methicillin-resistant (MRSA), prompting to switch the antimicrobial therapy to daptomycin at 8 mg/kg/day. This antibiotic was preferred over glycopeptides due to renal impairment with an estimated clearance of 30–35 mL/min and a hemodynamically unstable situation. The port and time of entry of this virulent pathogen remained unknown. Aspiration of the right hip, which was overtly inflamed, yielded pus with 108′400 leucocytes/μl and 91% polymorphonuclear cells. Cultures of the hip joint fluid confirmed the same strain of MRSA. Aspiration of the right knee was macroscopically not suspicious for infection, and the cell count was only at 160 leucocytes/μl. Both the hip and the knee presented a flexion contracture of more than 40°.

**Figure 1:**
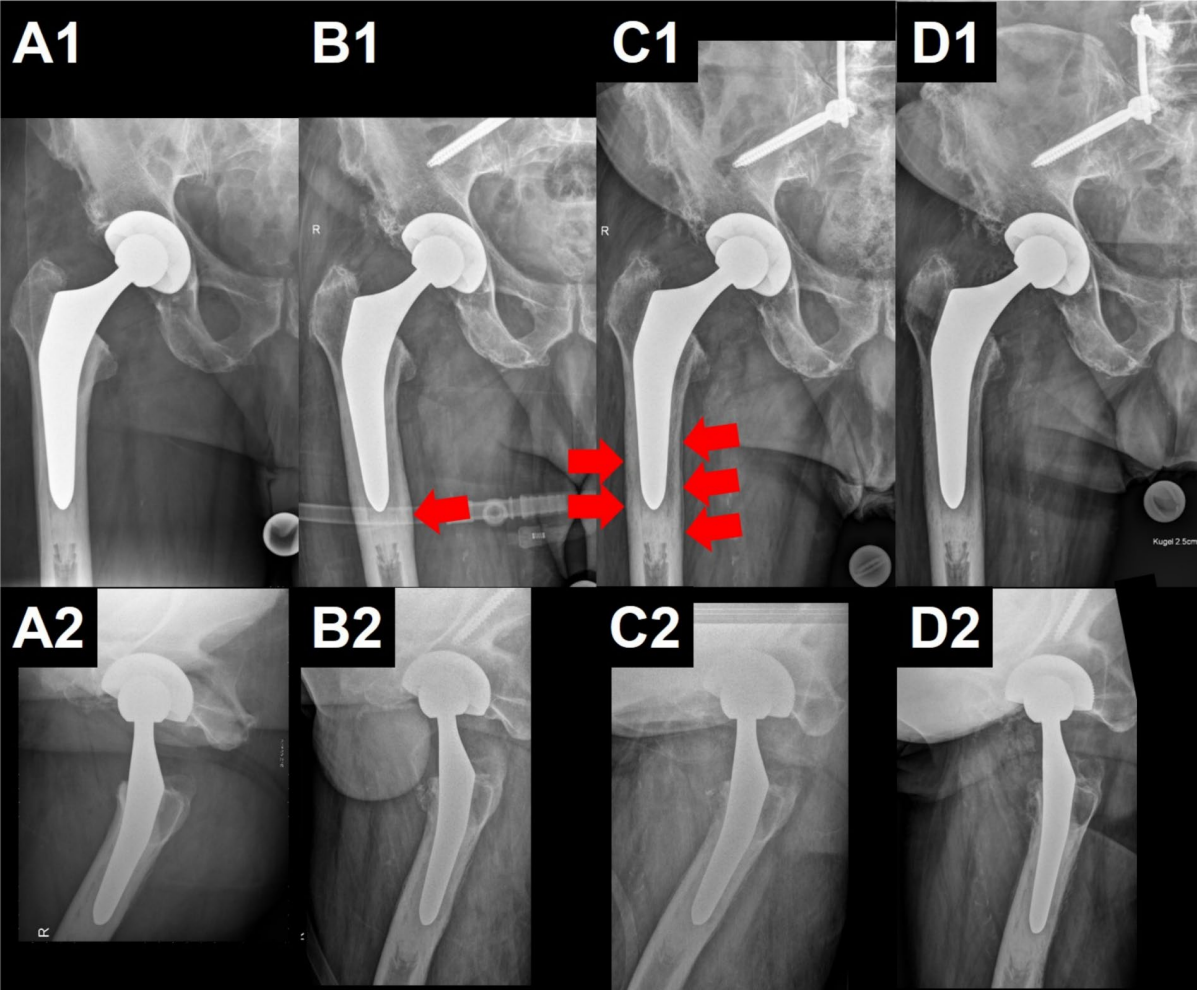
Radiographic follow-up of the patient described. The upper row shows the zone of interest of pelvic anteroposterior radiographs, illustrating the right hip. The lower row shows the corresponding axial views. Note the total hip arthroplasty (THA) with uncemented cup and cemented stem, as well as the partially illustrated material from the lumbopelvic fixation. In **(A)**, after THA. In **(B)**, regular follow-up 5 years after THA. Note the appearance of a cortical thickening at the tip of the stem (red arrow), most probably due to mechanical overload secondary to the line-to-line cementation. In **(C)**, 7 years after THA, when the patient was hospitalized due to the sepsis. Note the additional periosteal reaction (red arrows), which may still be caused by the above-mentioned mechanical issue and not necessarily by the infection. In **(D)**, the last radiographs, approximately 7 months after the revision. Technical differences in exposure of the radiograph explain differences in contrast, but no loosening is present.

Considering the confirmed patient’s desire for therapy, a rapidly positive response to the initial treatment, surgical treatment was aimed for on the third day to allow elimination of the oral anticoagulant. Shortly before surgery, cultures from the knee joint also yielded MRSA in low quantity. Considering the confirmed short duration of symptoms and as no component loosening was present, both joint replacements were therefore accessible to debridement and implant retention procedures. The alterations observed along the proximal femur could well be explained by mechanical issues associated with the line-to-line cementation of the stem (Figure 1) (18).

Surgical treatment began at the overtly more severely infected hip. Despite THA having been performed initially through an anterior approach, full exposure through the same approach was not feasible due to pronounced scar tissue formation around the deeply situated hip joint. The medial neocapsule remained inaccessible to debridement. Dislocation of the prosthesis was not possible, despite the use of a mobile traction table, rendering exchange of the modular components impossible. Finally, the surgery had to be aborted due to blood loss and increasing hemodynamic instability. Local application of antibiotic-loaded beads was not done due to the already poor renal failure and expected worsening following surgery.

Postoperatively, the patient stabilized rapidly. Considering the massive scar tissue formation around the hip, the infection was deemed chronic and the initial sepsis was most probably secondary. The PJI of the knee was likely secondary but had been diagnosed with a very low bacterial load. Considering age, comorbidities, as well as the surgical requirements for a cure of the PJI, the option of a suppressive antibiotic therapy was negotiated with the patient. He could be discharged back to his nursing facility on the 7^th^ postoperative day with an oral antibiotic suppression with trimethoprim/sulfamethoxazole.

The patient never had low back pain, and there never were any clinical signs of infection in the lumbar region. One of the infectious episodes of the last years treated as recurrent urinary tract infection was probably a *S. aureus* sepsis causing PJI of the hip, as postoperative infection had been ruled out thanks to the aspiration performed about 1.5 years post-THA.

**Figure 2:**
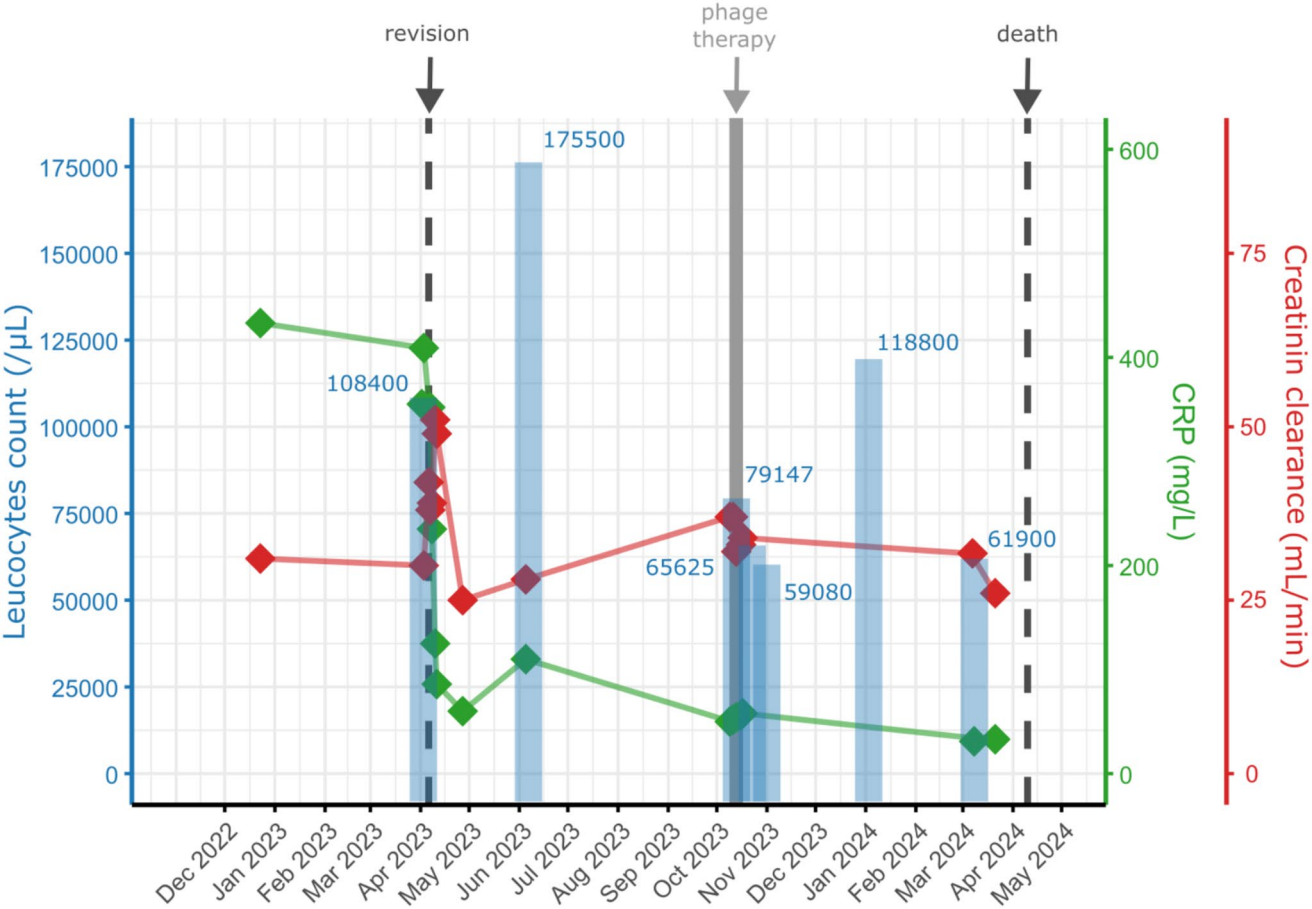
Overview of the serum CRP levels (green), the creatinine clearance (red) as estimated by the modification of diet in renal diseases (MDRD) formula, and of the leucocyte count from the hip joint aspirations (blue) over time from the patient described. Note the reduction of CRP following the phage treatment. Even if the leucocyte count from the joint aspiration remained elevated, there were no more clinical signs of infection and the bacterial growth in culture was notably delayed.

**Figure 3:**
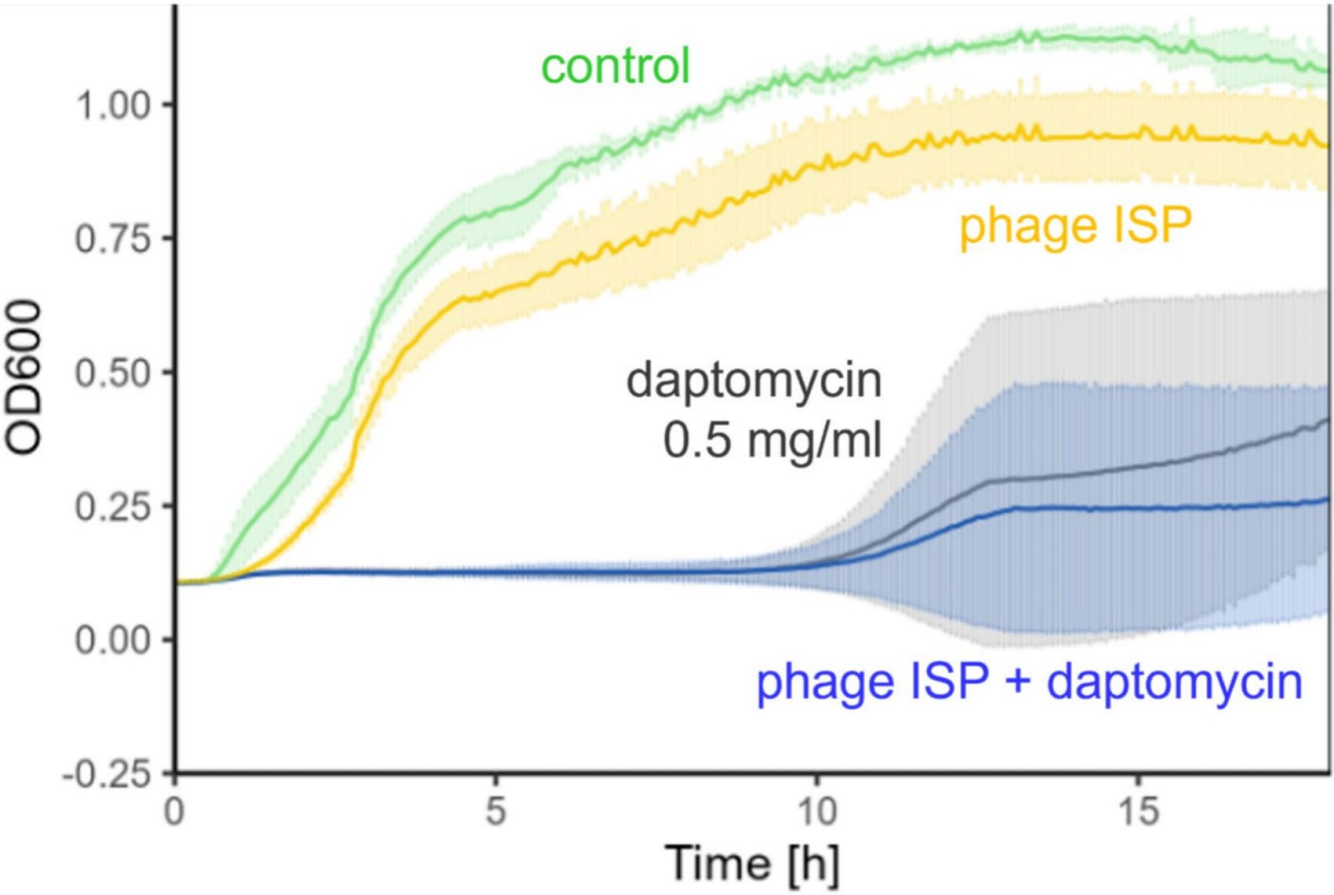
Phage susceptibility testing. Turbidity reduction assay of phage ISP on the patient’s MRSA strain as measured by optical density (OD 600 nm) over time (h) showing the effect of phage ISP (yellow; 10^7^ PFU/mL) and daptomycin (grey, 0.5 mg/L) alone and in combination (blue) on bacterial growth (control in green).

**Figure 4:**
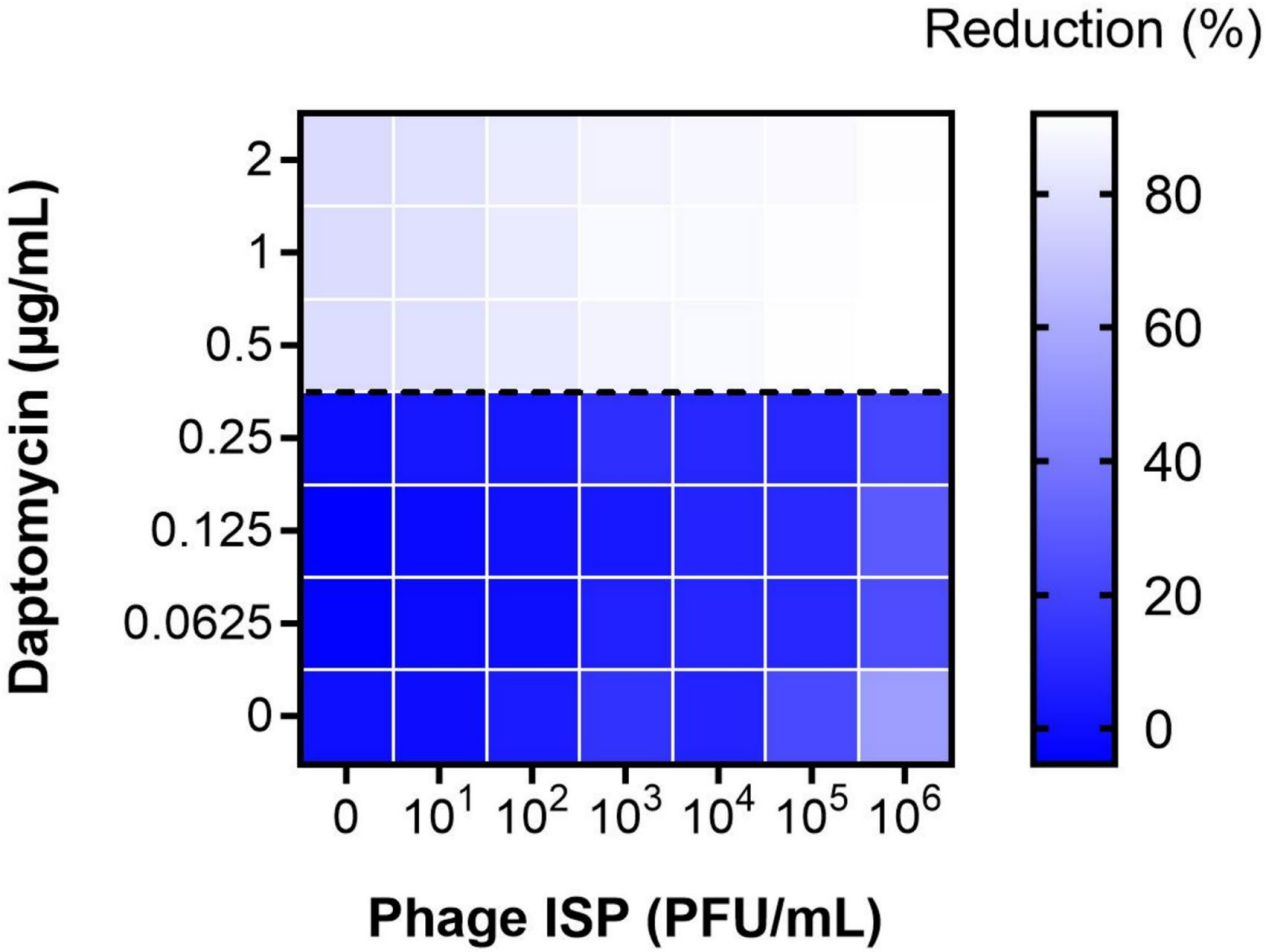
Heat map of phage-antibiotic synergy tested against the patient’s isolate at 12 h. Effect of phage ISP in combination with daptomycin as shown as the mean reduction of each combination; Reduction (%) = [(OD_growth control_ – OD_treatment_)/OD_growth control_] × 100. The dotted line indicates the minimum inhibitory concentration (MIC) of the strain to daptomycin. The graph was plotted using GraphPad Prism version 8.0.2 for Windows.

Over the following months, the patient remained pain-free, but the hip remained clinically inflamed, whereas the knee remained clinically inconspicuous. Serum C-reactive protein (CRP) levels stagnated initially but increased secondarily (Figure 2). As there were no treatment alternatives, particularly regarding oral suppression options, and as the patient still did not accept palliative treatment, this prompted investigations regarding possibilities for an adjunct treatment with bacteriophages. Bacteriophages were made available from the collection from Queen Astrid Military Hospital (Brussels, Belgium). Bacteriophage ISP had only a limited lytic activity against the MRSA strain of the patient (efficiency of plating: 0.1), but this was deemed sufficient as a small additive effect was observed *in vitro* in combination with daptomycin (Figure 3). Limited bacterial regrowth was observed after 10 h, which was not unexpected due to the use of rich growth media and was present to a lower degree when phage antibiotics were combined. The heat map of various concentrations of bacteriophages in combination with various concentrations of daptomycin is provided in Figure 4. Bacteriophage ISP is a frequently used staphylococcal phage in Magistral preparations at Queen Astrid Military Hospital and the specific batch underwent quality control by Sciesano for pH (mean 7.13; pass), identification (confirmed), microbial contamination (pass), and endotoxin quantification (<5 EU/mL).

The patient was hospitalized for this treatment about 6 months after the revision, as soon as the preparation could be made available, which took more than 3 months. Bacteriophages were applied parenterally as well as intraarticularly over a total of 10 days at a concentration of 10^7^ plaque forming units (PFU)/mL (Table 1). During the bacteriophage treatment, the oral antimicrobial therapy with trimethoprim/sulfamethoxazole was substituted with parenteral daptomycin at 8 mg/kg/day again. The patient tolerated the repeated joint infiltrations well. Joint aspirations were obtained prior to the start of phage therapy and then before subsequent doses. No bacteriophage was detected in the synovial fluid of either joint at the start of therapy, but was then detected in the synovial fluid of the hip prior to subsequent administrations (average 2×10^4^ PFU/mL), indicating prolonged presence at the site of infection. Low levels of MRSA were found in the hip synovial fluid throughout the treatment but no resistance to phage ISP was detected and there was no change in antibiotic susceptibility of the isolated MRSA strains. No more MRSA was detected in the knee.

**Table 1:** Overview of the administration scheme of the bacteriophages.

	Quantity\Day	1	2	3	4	5	6	7	8	9	10
Systemic	80 mL at 1 × 10^7^ PFU/mL in 100 mL saline over 6 h	X	X	X	X	X	X	X	X	X	X
Hip	20 mL at 1 × 10^7^ PFU/mL	X		X	X	X			X		
Knee	10 mL at 1 × 10^7^ PFU/mL	X							X		

A solution containing 10^7^ PFU/mL of the phage ISP was used. Bacteriophages were administered systemically as well as locally. For systemic application, 80 mL of phage ISP (1 × 10^7^ PFU/mL) was diluted in 100 mL of sterile saline and administered intravenously via a perfusor over a 6-h period, corresponding to an infusion rate of 30 mL/h. Intra-articular phage therapy was administered as a bolus injection. Under sterile conditions in a radiologic intervention suite, joint aspiration was performed under fluoroscopic guidance. Following the removal of synovial fluid, the phage ISP (20 mL at 1 × 10^7^ PFU/mL for the hip, 10 mL at 1 × 10^7^ PFU/mL for the knee) was injected into the joint space using a single-use syringe. Daptomycin was administered concomitantly during the 10-day course.

**Figure 5:**
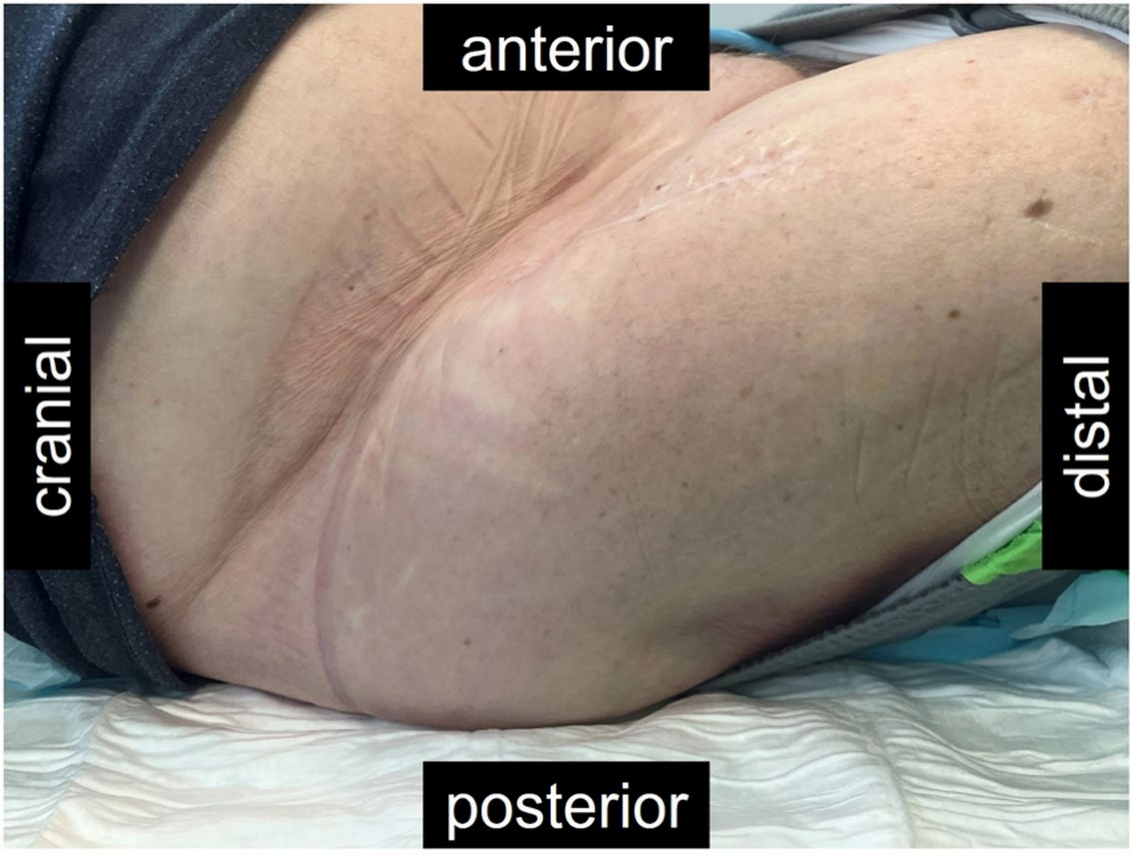
Picture of the right hip of the patient described, approximately 11 months after the revision, and 5 months after bacteriophage therapy. The scar from the anterior approach remained closed throughout. Fibrosis made the tissues around the hip hard and the hip remained fixated in the flexion illustrated. However, the patient had no more pain and the hyperemia nearly had disappeared after the treatment with bacteriophages.

The bacteriophage treatment was considered very successful by the patient. He noted an improvement of his general state and better mobility of his hip. Objectively, a disappearance of clinical signs of infection could be observed (Figure 5), as well as a regression of the CRP, even if a normalization was not reached (Figure 2). However, repeated aspirations of the hip joint proved persistence of the MRSA and leukocytes (Figure 2). Thus, the antimicrobial treatment with trimethoprim/sulfamethoxazole was maintained. Nevertheless, only small quantities of fluid could be aspirated, whereas initially large quantities of pus were present, and bacterial identification then required 5–8 days of culture.

The patient finally died in his nursing home about 6 months after the bacteriophage therapy, respectively 12 months after the revision, without presenting signs of infection or pain, although it was not possible to ascertain an ultimate cause of death. Obtaining follow-up samples to determine the presence of MRSA and bacteriophages, as well as phage neutralizing antibodies, was not possible due to the death of the patient shortly before the scheduled visit.

The patient had provided written informed consent for publication of anonymized data before the revision.

## Discussion

In the reported case bacteriophage therapy was considered due to persistent MRSA infection despite standard treatment, limited oral antibiotic options, and the patient’s comorbidities restricting further surgical interventions. Additionally, increased *in vitro* activity between daptomycin and the selected phage supported its use as a promising adjunctive strategy for infection control.

Classical surgical and antibiotic options may not be sufficiently effective or tolerable to treat successfully PJI, particularly when comorbidities limit therapeutic possibilities (4, 5, 7–11, 15, 19). Antibiotic suppression would be the classical option in case of treatment failure without surgical options (9, 12, 13). However, adequate drugs may not always be available or tolerated by the patient (4, 13), and antibiotic suppression may also not be sufficient to prevent overt recurrence of infection (12, 13, 20). As illustrated in this case, the administration of bacteriophages may be a valuable, less-invasive adjunct to successfully suppress the infection (12–14, 20). PJI persistence was proven by repeated aspirations (Figure 2). However, the bacteriophage-antibiotic synergy may have been decisive to obtain the desire control of infection. In this case, the bacteriophage therapy was tolerated without side effects.

While objective parameters showed only a marginal response (Figure 2), clinically and subjectively, there was a major benefit of the bacteriophage therapy (Figure 5). Such soft outcomes are difficult to document, particularly in retrospective studies. Differences in outcome parameters, however, have major implications on the reported treatment effectiveness in PJI (21, 22). The Musculoskelettal Infection Society provided guidelines for tiered outcome reporting to better assess treatment success (22). While this classification is not yet widely known, the bacteriophages would have transformed a Tier 3 or 4 (overt failures) into a Tier 2 outcome (successful control of the infection with antibiotic suppression) (22). Standardization of outcome reporting in the treatment of musculoskeletal infections is encouraged, particularly as functional outcomes and infection control may by more important than eradication of infection (14, 15, 21–25). This case suffered no adverse events from the bacteriophage administration. Minor and mostly local reactions to bacteriophage administration are reported only rarely (14, 15, 26).

Beyond the clinical benefit for this one patient, this report may be useful for the technical details made available regarding posology (Table 1), as this remains an open topic in bacteriophage therapy (14, 15). Bacteriophage selection, preparation and administration remains associated with major obstacles, greatly limiting availability and practicability. In this case, the procedure required more than 3 months from the timepoint of indication until the preparations were available. While selection, culture and preparation of the bacteriophages invariably takes a certain amount of time, the issue is primarily administrative, particularly when cross-country delivery is required (27). Particularly if more than one country is involved, which usually is necessary in Europe to access the various bacteriophage libraries available. Beyond the administrative burden, which is out of proportion for clinical care and which requires a dedicated research team, such delays may be inacceptable if the evolution of the infection dictates faster treatment. Nevertheless, research and developments in this domain should be pursued, particularly considering issues with future antibiotic limitations, costs associated with treatment failure in PJI, and considering the very interesting results published so far (14, 15). While bacteriophages are used so far only as last-resort salvage, this may have to be reconsidered to attribute a role earlier in the treatment algorithms.

Finally, the duration of symptoms is recommended as a decisive parameter to indicated prosthesis-retaining treatment (7–9). While the duration of symptoms was short, this patient overtly presented a chronic PJI of his hip. Despite impossibility to perform the treatment of choice, i.e., full revision of the components, infection control could be obtained, representing an acceptable option in such an elderly and multimorbid patient (7–9, 12, 13, 22–25).

## Data Availability

Raw data supporting the conclusions of theis article will be shared with researchers submitting a methodologically sound proposal. Requests should be directed to the corresponding author.
